# Association between cardiometabolic index and cardiovascular disease: evidence From the NHANES 2007–2018

**DOI:** 10.3389/fcvm.2025.1516591

**Published:** 2025-05-12

**Authors:** Feiyu Chen, Yongquan Niu, Runzhe Wu, Haodong Jiang, Jia Zhu, Congying Wang, Xiaojun Xia, Yunpeng Jin

**Affiliations:** Department of Cardiology, The Fourth Affiliated Hospital of School of Medicine, and International School of Medicine, International Institutes of Medicine, Zhejiang University, Yiwu, China

**Keywords:** cross-sectional study, cardiometabolic index, cardiovascular disease, NHANES, obesity

## Abstract

**Background:**

Although the cardiometabolic index (CMI) has gained recognition as a new tool for evaluating metabolic health, the relationship between CMI and cardiovascular disease (CVD) remains unclear. This research sought to explore the potential association between CMI and CVD.

**Methods:**

Participants from the 2007–2018 National Health and Nutrition Examination Survey (NHANES) were selected. Multivariable logistic regression analyses and smooth curve fitting were utilized to investigate this relationship, along with subgroup evaluations and interaction analyses.

**Results:**

This study included 12,837 subjects and the prevalence of CVD was 11.83%. After full adjustment, participants presenting with an increase of one unit in Ln-transformed CMI associated a 15% higher odds of CVD prevalence (OR = 1.15, 95% CI: 1.05–1.26). In the fully adjusted model, individuals falling into the highest CMI quartile (Quartile 4) demonstrated substantially 35% higher odds than those in the lowest CMI quartile (Quartile 1) (OR = 1.35, 95% CI: 1.11–1.66). In addition, there was no nonlinear relationship between CMI and CVD in our selected sample. This positive association was not greatly influenced by any of the stratifications.

**Conclusions:**

Among US adults, having higher CMI levels is substantially associated with higher odds of CVD prevalence. This finding suggests that regular monitoring of CMI levels could enable physicians to initiate early interventions, potentially slowing the progression of CVD. However, in order to corroborate our findings, further prospective investigations are still required.

## 1 Introduction

Worldwide, cardiovascular disease (CVD) continues to be one of the primary causes of death (1). Some studies reported that approximately 17.9 million deaths worldwide occur as a result of cardiovascular disease each year, making up 31% of global mortality (2, 3). These conditions are intimately linked to hyperlipidemia, hypertension, diabetes, and obesity (4, 5). The global burden of CVD remains substantial, especially in low-income nations and middle-income nations, despite CVD-related mortality has recently declined in high-income countries (6, 7). Therefore, preventing CVD and managing disease progression is critical.

The cardiometabolic index (CMI) has gained recognition as a new tool for evaluating metabolic health and predicting CVD risk (8). It not only explains the degree of obesity, but also provides a good indication of an individual's lipid levels (8, 9). CMI is calculated using triglyceride to high-density lipoprotein cholesterol ratio (TG/HDL-C ratio) and waist-to-height ratio (WHtR), which are considered strong indicators of an individual's metabolic status (10). Compared to other complex metabolic assessment tools, CMI offers a simpler calculation method, which enhances its potential application in clinical practice (11–13). Some research has explored the clinical relevance of cardiometabolic index in several different diseases, including hypertension (14), diabetes (15), left ventricular geometry change (16), and endometriosis (17).

As far as we know, although CMI offers important insights for diagnosing and predicting the risk of various metabolism-related diseases, research on the relationship between CMI and CVD is limited. Thus, using data from the National Health and Nutrition Examination Survey (NHANES) from 2007 to 2018, our research aims to further investigate the association between CMI and CVD.

## 2 Methods

### 2.1 Study population

This research utilized data from the NHANES, a continuous program designed to evaluate the dietary habits and health status of the U.S. population (18, 19). The survey follows a sophisticated sampling technique to ensure it represents the national population and is managed by the National Center for Health Statistics (NCHS). The NCHS Institutional Review Board approved the study ethically, with each volunteer providing their informed consent (20).

**Figure 1:**
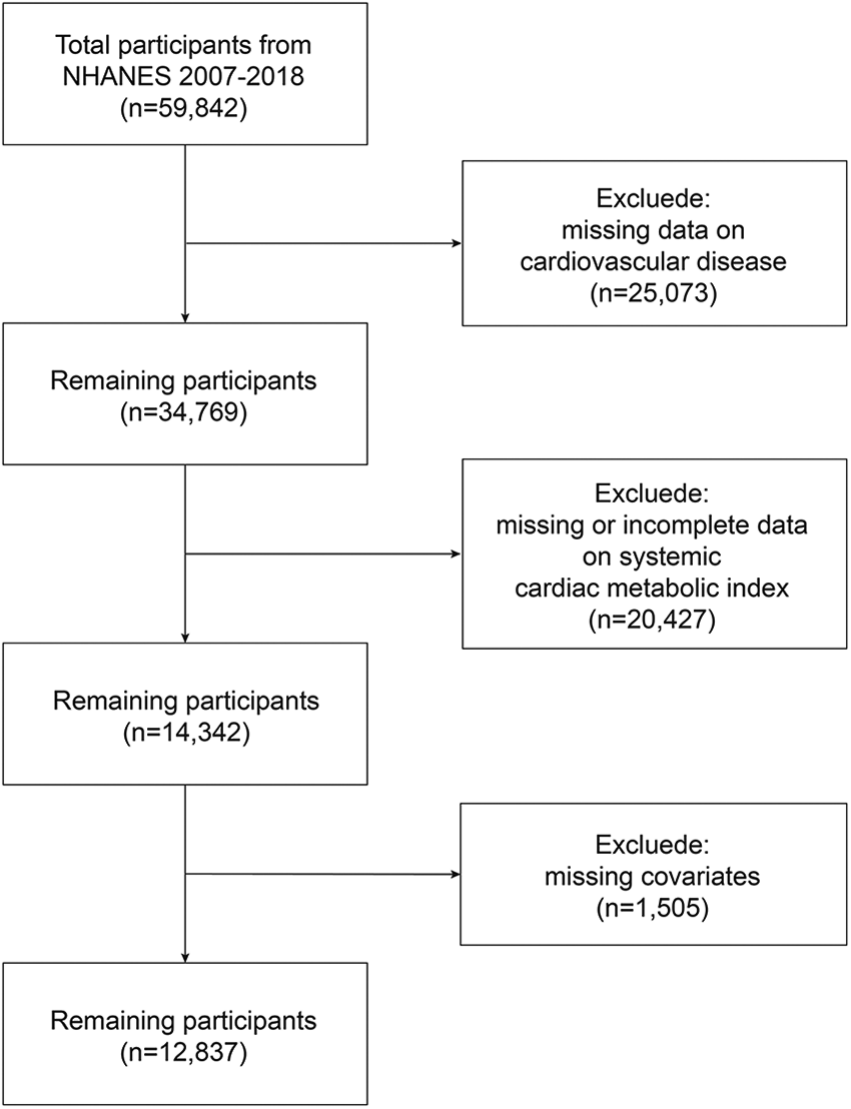
Flowchart of the sample selection from NHANES 2007–2018.

The study analyzed continuous NHANES data spanning 2007 to 2018, starting with 59,842 participants. Then, we excluded 25,073 participants with incomplete CVD evaluation data, 20,427 with missing complete data about CMI, and 1,505 whose covariate data were missing. 12,837 representative volunteers in all were eventually enrolled in this research (Figure 1).

### 2.2 CMI

The CMI for each participant is determined utilizing the following formula. CMI = [TG (mmol/L)/HDL-C (mmol/L)] × [WC (cm)/height(cm)].

### 2.3 Outcome ascertainment

The outcome variable for this study was CVD, which encompasses stroke, angina pectoris, congestive heart failure (CHF), heart attack, and coronary heart disease (CHD), based on previous research. “Has a doctor or other healthcare professional ever told you that you have coronary heart disease/stroke/heart attack/angina pectoris/congestive heart failure?” was the question posed to each individual. If an individual responded “yes” to any of the questions, they were identified as having CVD.

### 2.4 Covariables

The covariates considered in this study included age, weight, height, education level, income-to-poverty ratio, race, gender, waist circumference (WC), glycosylated hemoglobin (HbA1c), High-density lipoprotein cholesterol (HDL-C), body mass index (BMI), exercise status, smoking status, serum uric acid (SUA), triglycerides (TG), fasting blood glucose (FPG), serum creatinine (Scr), total cholesterol (TC), diabetes, hypertension, hyperlipidemia, serum phosphorus, serum calcium, vitamin D. The sporting status was determined by “engaged in moderate leisure activities”, and “smoked at least 100 cigarettes in life” indicated one's smoking status. “Have you ever been told you have diabetes, hypertension, or hyperlipidemia?” was the question used to gauge a person's history of diabetes, hypertension, and hyperlipidemia. You can access all of the data about these variables at https://www.cdc.gov/nchs/nhanes/.

### 2.5 Statistical analysis

Since the CMI is a skewed distribution, it was Ln-transformed by us to make it close to a normal distribution. Chi-square tests were utilized for categorical data and t-tests were employed for continuous variables to assess participant demographics, which were categorized based on CMI quartiles. Multivariate logistic regression analysis was used to examine the association between LnCMI and CVD. We built four models in total. Model 1 did not include any covariate adjustments. For Model 2, we took race, gender, and age into account. We made further adjustments for education level, income-to-poverty ratio, diabetes, smoker, exercise status in Model 3. In model 4, we additionally adjusted for hypertension, hyperlipidemia, TC, serum calcium, serum phosphorus, Scr, SUA, HbA1c, vitamin D. Stratified analyses and interaction tests were also conducted by race, age (<30/≥30, <40/≥40, <50/≥50, <60/≥60, <70/≥70 years), exercise (yes/no), smoking (yes/no), gender (male/female), hypertension (yes/no), diabetes mellitus (yes/no) and hyperlipidemia (yes/no). To ascertain the nonlinear association between CMI and CVD, smoothing curve fitting was implemented additionally. All statistical analyses were conducted utilizing EmpowerStats (version 2.0) and R (version 4.3). The definition of statistical significance is *p* < 0.05 on both sides.

## 3 Results

### 3.1 Baseline characteristics

Table 1 displays the characteristics of the research population. In this study, 12,837 participants were enrolled, with an average age of 51.03 ± 17.25 years. Of these, 51.94% were female and 48.06% were male. The ranges of LnCMI in transform for quartiles 1‒4 were −3.61 to −1.25 (≤−1.25), −1.25 to −0.68 (≤−0.68), −0.68 to −0.11 (≤−0.11) and −0.11‒3.68 (≤3.68), respectively. Overall, 11.83% of individuals had CVD, and this frequency increased as the CMI quartiles increased (Quartile 1: 7.29%, Quartile 2: 11.28%, Quartile 3: 12.12%, Quartile 4: 16.59%; *P* < 0.001). The distribution of the following variables showed significant differences between quartiles: age, race, gender, income-to-poverty ratio, education, waist circumference, BMI, moderate activity, smoking habits, hyperuricemia, hypertension and diabetes, HDL-C, serum uric acid, serum creatinine, total cholesterol, HbA1c, triglycerides and Vitamin D (all *P* < 0.05). Individuals in the higher CMI group, compared to those in the lowest CMI group, had higher BMI, WC, SUA, TC, TG, and HbA1c levels, and lower Vitamin D and HDL-C levels, along with an increased likelihood of hyperuricemia, diabetes, and hypertension (all *P* < 0.05). Notably, there was a greater prevalence of CVD among individuals with high CMI values (*P* < 0.05).

**Table 1:** Baseline characteristics of study population according to cardiometabolic index quartiles.

Characteristics	Cardiometabolic index				*P*-value
	Q1 (*N* = 3,196)	Q2 (*N* = 3,209)	Q3 (*N* = 3,219)	Q4 (*N* = 3,213)	
Age (years)	47.08 ± 18.01	51.43 ± 17.62	52.82 ± 16.93	52.76 ± 15.76	<0.001
Gender, (%)					<0.001
Male	37.64	45.78	50.05	58.70	
Female	62.36	54.22	49.95	41.30	
Race/ethnicity, (%)					<0.001
Mexican American	8.14	12.34	16.59	18.46	
Other Hispanic	7.76	10.47	12.77	12.54	
Non-Hispanic White	38.89	41.07	39.61	47.93	
Non-Hispanic Black	29.19	24.06	18.02	9.96	
Other races	16.02	12.06	13.02	11.11	
Education level, (%)					<0.001
<high school	16.27	21.50	26.00	29.35	
High school	19.87	22.03	22.52	23.31	
>high school	63.86	56.47	51.48	47.34	
PIR	2.68 ± 1.60	2.59 ± 1.57	2.48 ± 1.53	2.32 ± 1.50	<0.001
Waist circumference (cm)	88.05 ± 12.76	97.92 ± 14.28	103.83 ± 14.67	109.85 ± 15.19	<0.001
Weight (kg)	69.88 ± 16.19	79.66 ± 19.20	85.53 ± 20.75	92.48 ± 22.17	<0.001
Height (cm)	166.62 ± 9.44	167.11 ± 10.18	166.80 ± 10.23	167.74 ± 10.37	<0.001
BMI (kg/m^2^)	25.11 ± 5.21	28.46 ± 6.20	30.66 ± 6.57	32.76 ± 6.82	<0.001
Moderate activities, (%)					<0.001
Yes	49.16	42.75	38.43	33.99	
No	50.84	57.25	61.57	66.01	
Smoker, (%)					<0.001
Yes	36.95	41.13	45.29	52.35	
No	63.05	58.87	54.71	47.65	
Diabetes, (%)					<0.001
Yes	6.01	10.88	17.05	23.09	
No	93.99	89.12	82.95	76.91	
Hypertension, (%)					<0.001
Yes	27.13	37.11	42.22	50.39	
No	72.87	62.89	57.78	49.61	
High cholesterol, (%)					<0.001
Yes	23.72	35.00	41.22	50.08	
No	76.28	65.00	58.78	49.92	
HDL-c (mmol/L)	1.80 ± 0.42	1.47 ± 0.30	1.27 ± 0.25	1.04 ± 0.21	<0.001
TC (mmol/L)	4.74 ± 0.97	4.86 ± 1.02	4.98 ± 1.09	5.25 ± 1.18	<0.001
TG (mmol/L)	0.64 ± 0.20	0.98 ± 0.24	1.38 ± 0.33	2.62 ± 1.94	<0.001
Serum creatinine (mg/dl)	0.86 ± 0.42	0.89 ± 0.48	0.89 ± 0.39	0.93 ± 0.47	<0.001
Serum uric acid (μmol/L)	288.95 ± 73.81	317.60 ± 78.52	339.42 ± 83.01	365.04 ± 88.78	<0.001
Serum calcium (mmol/L)	2.34 ± 0.09	2.33 ± 0.09	2.33 ± 0.09	2.34 ± 0.09	0.099
Serum phosphorus (mmol/L)	1.20 ± 0.17	1.18 ± 0.17	1.17 ± 0.18	1.17 ± 0.18	<0.001
HbA1c (%)	5.48 ± 0.70	5.69 ± 0.94	5.92 ± 1.19	6.18 ± 1.38	<0.001
Vitamin D (nmol/L)	67.50 ± 29.90	66.76 ± 29.20	64.82 ± 27.77	63.45 ± 24.92	<0.001
CVD, (%)					<0.001
Yes	7.29	11.28	12.12	16.59	
No	92.71	88.72	87.88	83.41	

Mean ± SD for continuous variables: the *P* value was calculated by the linear regression model; (%) for categorical variables: the *P* value was calculated by the chi-square test.

PIR, the ratio of income to poverty; BMI, body mass index; Q, quartile; HDL-C, high-density lipoprotein cholesterol; TC, total cholesterol; TG, triglycerides; HbA1c, glycosylated hemoglobin; CVD, cardiovascular disease.

Additionally, we have plotted a Correlation Heatmap (Supplementary Figure S2) to analyze the linear relationships among six variables: CMI, WC, height, HDL-C, TG, and BMI. The results demonstrate a strong positive correlation between CMI, TG, WC, and BMI, while HDL-C exhibits a negative correlation with both CMI and TG.

### 3.2 Association between CMI and CVD

**Figure 2:**
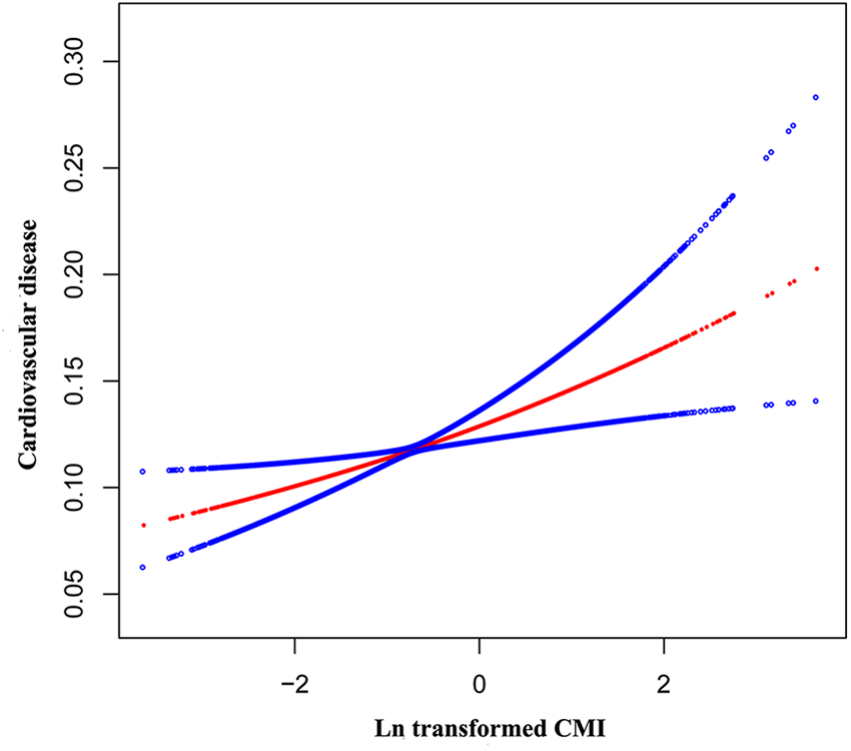
The nonlinear associations between Ln transformed CMI and CVD. The solid red line represents the smooth curve fit between variables. Blue bands represent the 95% of confidence interval from the fit.

Table 2 shows that across all four adjusting models, CMI was positively associated with CVD. In model 1, each unit increase in Ln-transformed CMI was associated with a 45% rise in the odds of CVD prevalence (OR = 1.45, 95% CI: 1.36–1.54). After full adjustment, participants presenting with an increase of one unit in Ln-transformed CMI associated a 15% higher odds of CVD prevalence (OR = 1.15, 95% CI: 1.05–1.26). Besides, the statistical significance of this association persisted even after treating LnCMI as quartiles. Individuals falling into the highest CMI quartile (Quartile 4) demonstrated substantially 35% higher odds than those in the lowest CMI quartile (Quartile 1) (OR = 1.35, 95% CI: 1.11–1.66) (*P* for trend = 0.006). Additionally, The nonlinear association between CMI and CVD was further explored by employing a smooth curve fitting technique, as illustrated in Figure 2. Our findings suggest that there was no nonlinear relationship between CMI and CVD in our selected sample.

**Table 2:** Association of CMI with CVD in different models among all participants.

Ln (CMI)	Model 1	Model 2	Model 3	Model 4
	OR (95%CI)	OR (95%CI)	OR (95%CI)	OR (95%CI)
Continuous	1.45 (1.36,1.54)	1.49 (1.38, 1.60)	1.14 (1.06, 1.24)	1.15 (1.05, 1.26)
Quartile				
Q1	1 (ref)	1 (ref)	1 (ref)	1 (ref)
Q2	1.62 (1.36, 1.92)	1.36 (1.13, 1.63)	1.18 (0.97, 1.42)	1.12 (0.92, 1.36)
Q3	1.75 (1.48, 2.08)	1.46 (1.21, 1.75)	1.08 (0.89, 1.30)	1.02 (0.84, 1.24)
Q4	2.53 (2.15, 2.98)	2.36 (1.97, 2.82)	1.40 (1.16, 1.70)	1.35 (1.11, 1.66)
*P* for trend	<0.001	<0.001	<0.001	0.006

Model 1: no covariates were adjusted. Model 2: age, gender, and race were adjusted. Model 3: age, gender, race, education level, PIR, exercise status, smoking status, diabetes were adjusted. Model 4: age, gender, race, education level, PIR, exercise status, smoking status, diabetes, hypertension, hyperlipidaemia, serum calcium, serum phosphorus, Scr, SUA, HbA1c, vitamin D were adjusted.

PIR, the ratio of income to poverty; Q, quartile; Scr, serum creatinine; SUA, serum uric acid; HbA1c, glycosylated hemoglobin; CMI, cardiometabolic index; CVD, cardiovascular disease.

Since HS CRP data in NHANES is only available for the years 2015–2018, we conducted a separate statistical analysis (Supplementary Table S1) specifically for this period.

### 3.3 Subgroup analyses

**Figure 3:**
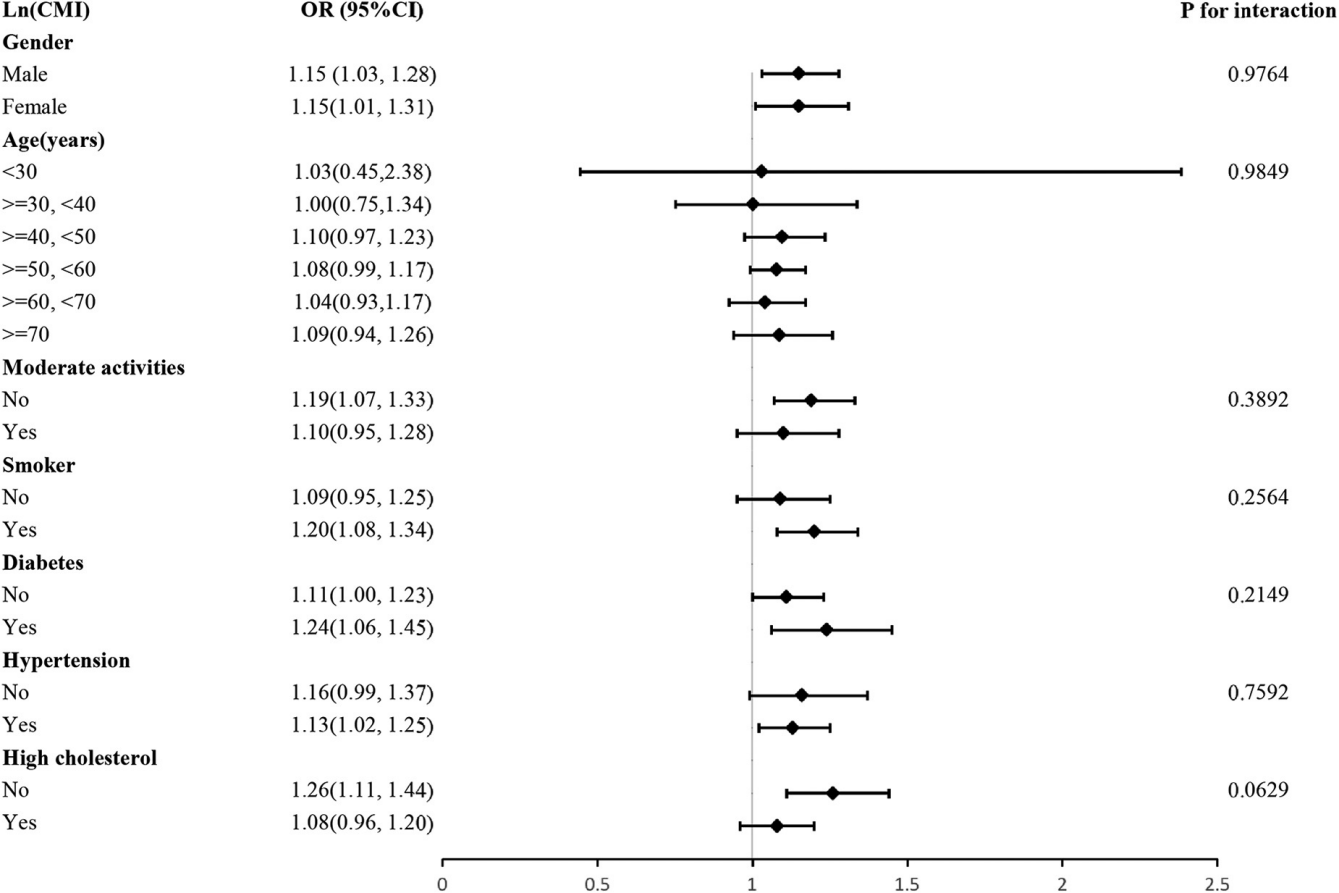
Subgroup and interaction analysis of LnCMI and CVD by gender, age, moderate activities, smoker, hypertension, diabetes and hyperlipidemia.

We investigated whether the association between LnCMI and CVD was robust in different demographic settings by performing a subgroup analysis with an interaction test. Figure 3 demonstrates that for every unit rise in LnCMI, there is a 15% higher odds of CVD prevalence in both males (OR = 1.15, 95% CI: 1.03–1.28) and females (OR = 1.15, 95% CI: 1.01–1.31). Gender had no significant effect on the relationship between LnCMI and CVD, according to the interaction analysis (*P* for interaction = 0.9764). Additionally, the positive association found between LnCMI and CVD was not greatly influenced by any of the stratifications, such as gender, exercise status, smoking status, diabetes, hyperlipidemia, age, or hypertension (all *P* for trend >0.05).

## 4 Discussion

We observed that individuals with higher CMI had higher odds of CVD prevalence in our cross-sectional study, which included 12,837 participants. There was shown to be a positive linear connection between CMI and CVD that remained robust when all variables were fully adjusted for. Additionally, this association remained consistent across various demographic settings. Our findings provide evidence suggesting that CMI may predict the occurrence of cardiovascular disease. Furthermore, we plotted ROC curve (Supplementary Figures S1, S3, S4) to illustrate that CMI is applicable to both diabetic and non-diabetic populations and is a better predictor of cardiovascular disease than metabolic syndrome.

Based on previous studies, obesity is a definite factor in the risk of CVD and mortality among adults (21, 22). Although prior research has indicated an association between the odds of CVD prevalence and traditional obesity-related indices like BMI (23–25), the obesity paradox implies that BMI and other general body fatness metrics are not capable of assessing the distribution of body fat or individual's metabolic health (26, 27). CMI, which includes WHtR and TG/HDL-C ratio, better reflects the interaction between body fat distribution and metabolic function than traditional obesity-related indicators (8). BMI, in comparison to CMI, is calculated solely based on weight and height and does not accurately reflect fat distribution or metabolic health status. It fails to distinguish between muscle and fat and has a weaker predictive capability for abdominal obesity and metabolic abnormalities. WC, while a direct measure of abdominal obesity, still has its limitations when used in isolation. It cannot differentiate between metabolically healthy obesity and metabolically unhealthy obesity. The diagnosis of metabolic syndrome encompasses TG, fasting blood glucose,blood pressure, WC, and HDL-C, which involves relatively cumbersome data collection and is prone to significant information gaps. CMI, on the other hand, can identify high-risk individuals earlier and more accurately, without the need to wait for the fulfillment of three metabolic abnormality criteria to assess risk. Research has shown that WHtR is a more effective indicator of the risk of stroke and CHD than BMI and WC (28, 29). Compared to other single indicators, the TG/HDL-C ratio is more closely linked to obesity, particularly with visceral fat accumulation, making it a better indicator for assessing metabolic health (30, 31). Clinicians can modify treatment plans in response to fluctuations in this ratio, aiming to lower the risk of cardiovascular events (32, 33). To our knowledge, several studies have shown a positive relationship between CMI and the odds of CVD prevalence, but these findings have thus far been validated only in East Asian populations (14, 16, 34, 35). For example, Wang et al. discovered a significant and independent relationship between female ischemic stroke and CMI, In a cross-sectional study involving 11,345 Chinese individuals. Moreover, Ichiro et al. also reported that CMI serves as a valuable indicator of how atherosclerosis is progressing in PAD patients and may even be able to predict the patient's prognosis. This research further validates the relationship between CMI and CVD among American adults.

Various underlying mechanisms might account for this relationship. First, in obese individuals, increased adipose tissue leads to a significant increase in leptin secretion (36). Leptin is a versatile adipokine. In addition to its metabolic functions, leptin exhibits potent pro-inflammatory effects that play a significant part in cardiovascular disease development. A notable function of leptin within the vascular system is its ability to enhance the interaction between endothelial cells and monocytes (37). Monocytes traverse the vascular endothelium through the action of adhesion molecules (38). These monocytes then cross the endothelial layer, transform into macrophages, and take up low-density lipoproteins (LDL) to form foam cells that eventually form atherosclerotic plaques (39). TNF-α released by macrophages also induces vascular endothelial cells to produce a substantial quantity of reactive oxygen species (ROS), and these oxidized substances damage endothelial cells and inhibit their release of nitric oxide (NO), leading to impaired vasodilatation (40, 41). Second, compared to general body fat, visceral adipose tissue (VAT) cells are relatively active and are pivotal in the development of CVD due to their release of MCP-1, IL-6, and TNF-α (42, 43). These molecules foster a persistent, low-grade inflammatory state within VAT, leading to dysfunction within the adipose tissue (44, 45). VAT accumulation elevates circulating free fatty acids, aggravating dyslipidemia and intensifying metabolic imbalances (46, 47). Elevated free fatty acids (FFA) in the liver lead to the buildup of intracellular lipid metabolites like diacylglycerol and ceramides (48). These metabolites disrupt the function of insulin receptor substrate (IRS), thereby hindering insulin's ability to suppress gluconeogenesis (49). Moreover, the inflammatory environment also disrupts glucose regulation, promoting insulin resistance and ultimately leading to type 2 diabetes (50, 51). Collectively, these factors substantially heighten the odds of cardiovascular events (52).

The key advantage of this research is that we used NHANES data, which has a large and nationally representative sample size. In addition, we accounted for potential confounding variables to enhance the reliability of our findings. Finally, we further validated our results with subgroup analyses in different populations. Nevertheless, we admit that our study has certain limitations. First, despite adjusting for numerous potential covariates, it was not possible to completely rule out the effects of unidentified or unmeasured confounders. For instance, the medication status of patients is indeed a significant covariate; however, the NHANES database presented substantial gaps in the relevant data regarding patient medication, rendering its inclusion unfeasible. Second, it is impossible for us to infer a causal relationship between CMI and CVD because of the cross-sectional design. Third, the sample for this study consisted solely of a US population.

## 5 Conclusion

Our research revealed that among US adults, having higher CMI levels is substantially associated with higher odds of CVD prevalence. Therefore, we propose CMI as a potential biomarker of CVD in US individuals. However, in order to corroborate our findings, further prospective investigations are still required.

## Data Availability

The raw data supporting the conclusions of this article will be made available by the authors, without undue reservation.
