# Supplementary material for: Association between cardiometabolic index and cardiovascular disease: evidence From the NHANES 2007–2018
Source: Front Cardiovasc Med. 2025 May 12;12:1516591. doi: 10.3389/fcvm.2025.1516591 (PMC12104185; doi:10.3389/fcvm.2025.1516591)

Supplementary Material

Supplementary Table 1: Association of CMI with CVD in different models among all participants.

Supplementary Figure 1: Comparison of CMI and Metabolic Syndrome for Predicting Cardiovascular Disease by ROC Curve Analysis.

Supplementary Figure 2: The correlation heatmap of CMI, WC, height, HDL-C, TG and BMI.

Supplementary Figure 3: ROC Curve Analysis of CMI for Cardiovascular Disease Prediction in Diabetic Population.

Supplementary Figure 4: ROC Curve Analysis of CMI for Cardiovascular Disease Prediction in Non-diabetic Population.

This supplementary material has been provided by the authors to give readers additional information about their work.

Supplementary Table 1: Association of CMI with CVD in different models among all participants.

Ln(CMI)	Model 1<br>OR (95%CI)	Model 2<br>OR (95%CI)	Model 3<br>OR (95%CI)	Model 4<br>OR (95%CI)
Continuous	1.40 (1.26, 1.56)	1.47 (1.30, 1.66)	1.15 (1.01, 1.32)	1.16 (1.00, 1.35)
Quartile				
Q1	1(ref)	1(ref)	1(ref)	1(ref)
Q2	1.80 (1.34, 2.43)	1.62 (1.18, 2.23)	1.39 (1.00, 1.93)	1.38 (0.99, 1.94)
Q3	1.92 (1.43, 2.58)	1.65 (1.20, 2.27)	1.16 (0.83, 1.61)	1.15 (0.81, 1.62)
Q4	2.63 (1.98, 3.49)	2.76 (2.02, 3.77)	1.62 (1.17, 2.26)	1.60 (1.12, 2.29)
P for trend	<0.001	<0.001	0.013	0.029

Model 1: no covariates were adjusted. Model 2: age, gender, and race were adjusted. Model 3: age, gender, race, education level, PIR, exercise status, smoking status, diabetes were adjusted. Model 4: age, gender, race, education level, PIR, exercise status, smoking status, diabetes, hypertension, hyperlipidaemia, serum calcium, serum phosphorus, Scr, SUA, HbA1c, vitamin D, HS CRP were adjusted. Abbreviation: PIR, the ratio of income to poverty; Q, quartile; Scr, serum creatinine; SUA, serum uric acid; HbA1c, glycosylated hemoglobin; CMI, cardiometabolic index; CVD, cardiovascular disease; HS CRP, HS C-Reactive Protein.

Supplementary Figure 1:

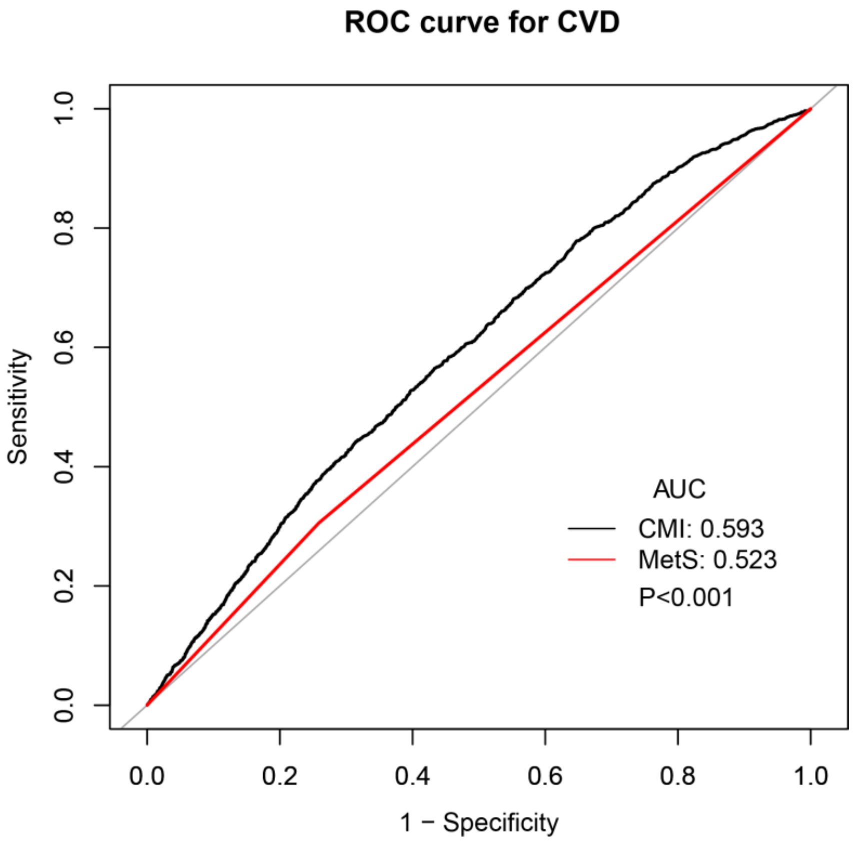

Supplementary Figure 2:

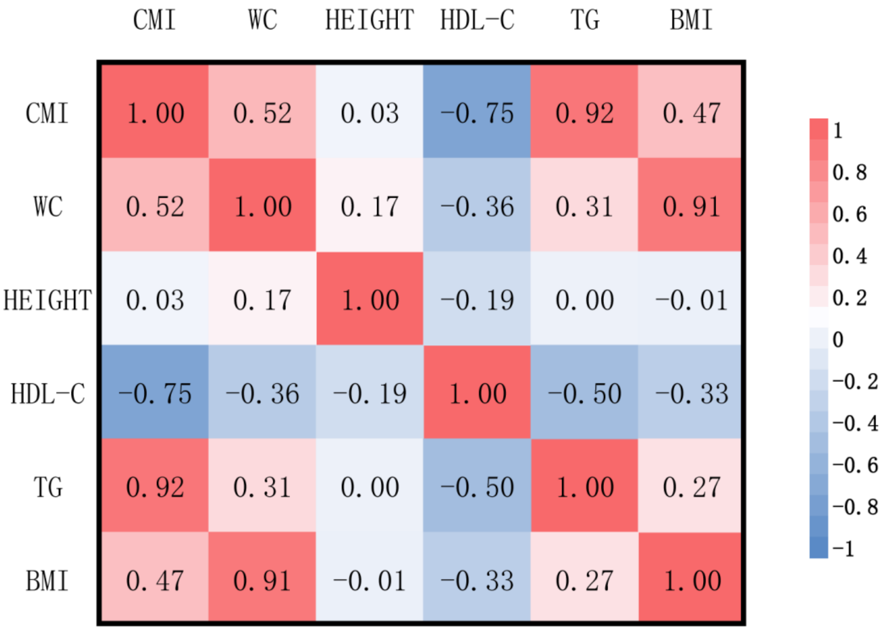

Supplementary Figure 3:

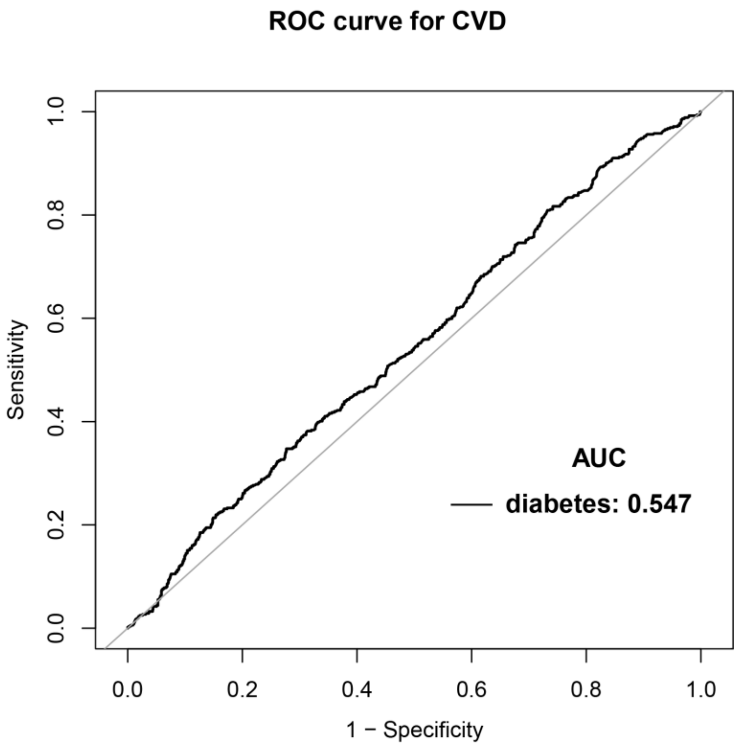

Supplementary Figure 4:

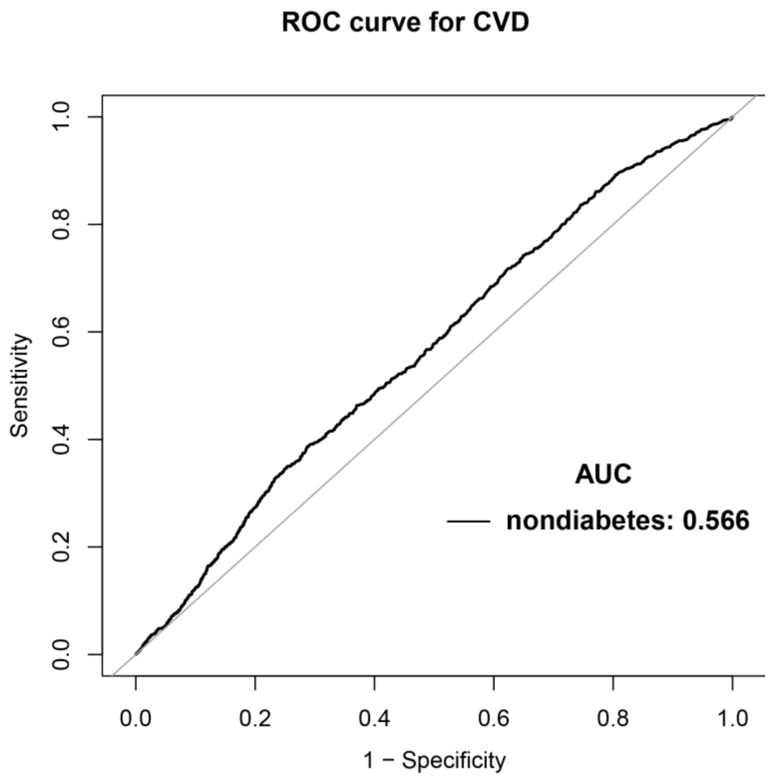

Supplement: Supplementary file 1 [file Datasheet1.pdf]
