# Supplementary figures and images for: Association between cardiometabolic index and cardiovascular disease: evidence From the NHANES 2007–2018
Source: Front Cardiovasc Med. 2025 May 12;12:1516591. doi: 10.3389/fcvm.2025.1516591 (PMC12104185; doi:10.3389/fcvm.2025.1516591)

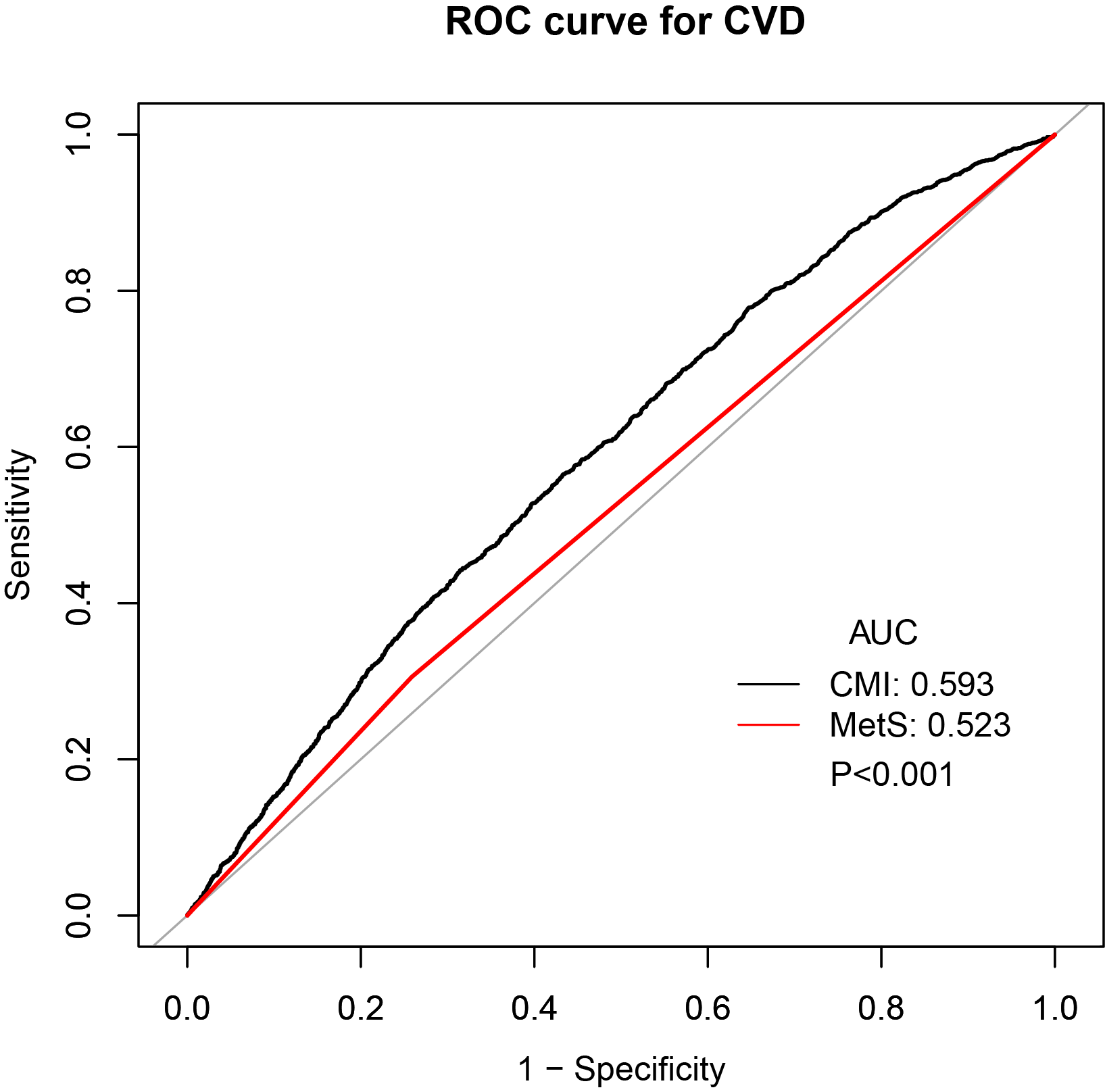

Supplement: Supplementary file 2 [file Image1.tif]

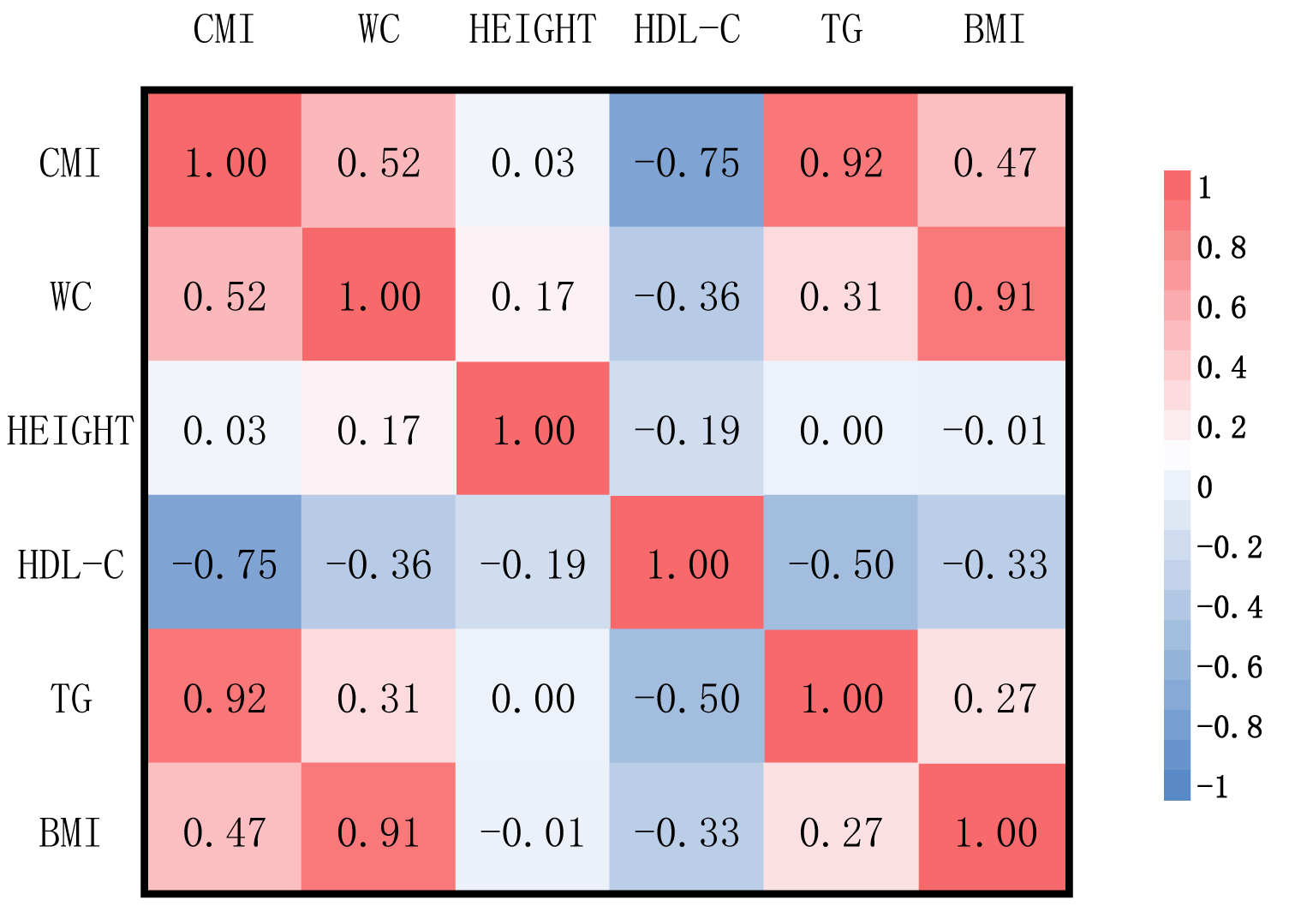

Supplement: Supplementary file 3 [file Image2.tif]

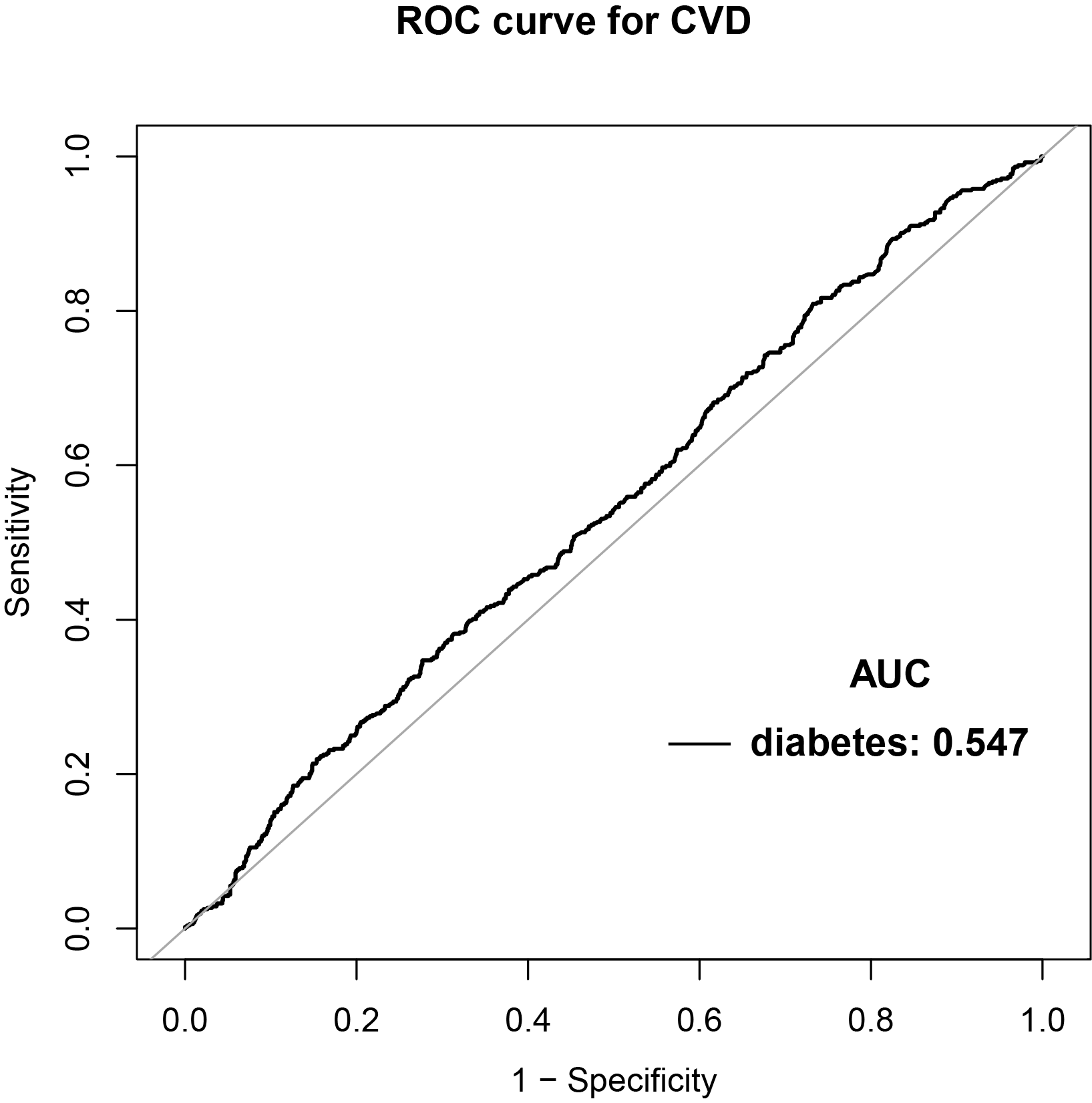

Supplement: Supplementary file 4 [file Image3.tif]

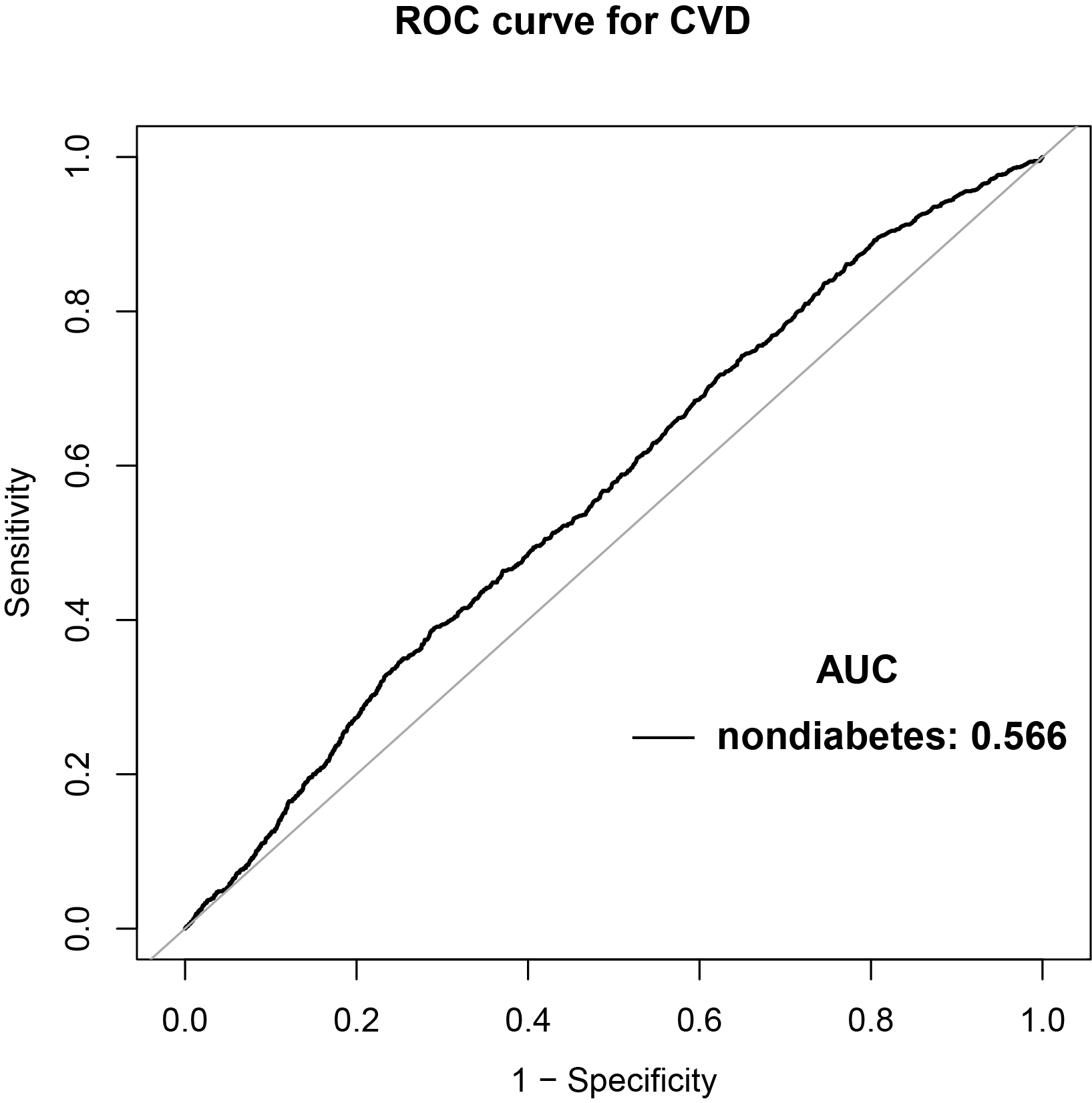

Supplement: Supplementary file 5 [file Image4.tif]
